# Passion fruit-like nano-architectures: a general synthesis route

**DOI:** 10.1038/srep43795

**Published:** 2017-03-03

**Authors:** D. Cassano, J. David, S. Luin, V. Voliani

**Affiliations:** 1 Center for Nanotechnology Innovation @NEST, Istituto Italiano di Tecnologia, P.zza San Silvestro, 12 - 56126, Pisa (PI), Italy; 2 NEST, Scuola Normale Superiore, P.zza San Silvestro, 12 - 56126, Pisa (PI), Italy; 3 NEST, Istituto Nanoscienze – CNR, P.zza San Silvestro, 12 - 56126, Pisa (PI), Italy

## Abstract

Noble metal nanostructures have demonstrated a number of intriguing features for both medicine and catalysis. However, accumulation issues have prevented their clinical translation, while their use in catalysis has shown serious efficiency and stability hurdles. Here we introduce a simple and robust synthetic protocol for passion fruit-like nano-architectures composed by a silica shell embedding polymeric arrays of ultrasmall noble metal nanoparticles. These nano-architectures show interesting features for both oncology and catalysis. They avoid the issue of persistence in organism thanks to their fast biodegradation in renal clearable building blocks. Furthermore, their calcination results in yolk-shell structures composed by naked metal or alloy nanospheres shielded from aggregation by a silica shell.

The interest on noble metal nanoparticles (NPs) relies on their peculiar optical, physical and biological behaviour^1^. Among them, gold is the most investigated metal for health applications owing to its biocompatibility, surface modifiability, and visible-NIR optical response^2^,^3^,^4^. Nevertheless, other noble metal NPs play a pivotal role in nanomedicine. For example, silver NPs have been intensively exploited in cancer therapy, treatment of viral diseases, and as antibacterial tools^5^,^6^. Furthermore, platinum NPs have shown Reactive Oxygen Species (ROS) scavenging properties in a wide range of vascular diseases^5^,^7^. However, among the number of proposed metal nanoparticles, none of them have arrived yet to the market^8^. One of the major concerns in clinical translation of metal nanoparticles is related to the their persistence in organisms^9^,^10^. Indeed, the common fate for metal NPs bigger than 10 nm is accumulation in liver and spleen^11^. On the other hand, NPs smaller than 5 nm can be effectively cleared by the renal pathway, but their functionality might be lost or severely altered, even if some of them still maintain optimal physiological properties^11^,^12^,^13^.

Metal nanoparticles have also attracted interest for their possible catalytic activity over a wide range of reactions^14^. For example Ag NPs have been successfully exploited as catalytic tools for reduction of aromatic compounds^15^ while Au, Pt and bimetallic Au/Pt NPs have demonstrated promising efficiency as nanocatalysts in oxygen reduction reactions and treatment of automotive exhaust gas^16^,^17^. Unluckily, their widespread diffusion is hampered mainly by the two following conflicting hurdles: i) the absence of a coating on the NPs surface increases their efficiency but causes their aggregation, resulting in a dramatic decrease of their catalytic performance and recycling, and ii) the presence of stabilizing agents enveloping NPs avoids their aggregation but severely compromises their catalytic activity by reducing the accessible surface area^18^.

Thus: i) translation of nanotechnologies in medicine needs to combine the feasibility of body clearance typical of ultrasmall NPs with the physical behaviour of bigger NPs^10^, and ii) metal NPs based catalysis can be effective only if reactive naked metal surfaces and colloidal stability are provided jointly^19^.

In this regard, we have recently introduced a 100 nm biodegradable passion fruit-like nano-architecture in which 3 nm gold NPs are strictly packed in polymer arrays surrounded by a silica shell^20^. We demonstrated that these nano-architectures can: i) mimic the optical behaviour of 30 nm gold NPs, and ii) be completely biodegraded in 48 h in cellular environment into potentially kidney-clearable building blocks^21^. Additionally, their calcination produces a yolk-shell nano-architecture composed by naked 20 nm gold NPs inside a stabilizing silica shell^20^. Despite other nanomaterials may share similar structure with passion fruit-like nano-architectures, the synthetic protocol and the final behaviours are not overlapping^22^.

Here we demonstrate the versatility of our synthetic approach by generalizing our protocol to other noble metals, in particular platinum, silver and gold/platinum mixtures.

## Results and Discussion

**Figure 1:**
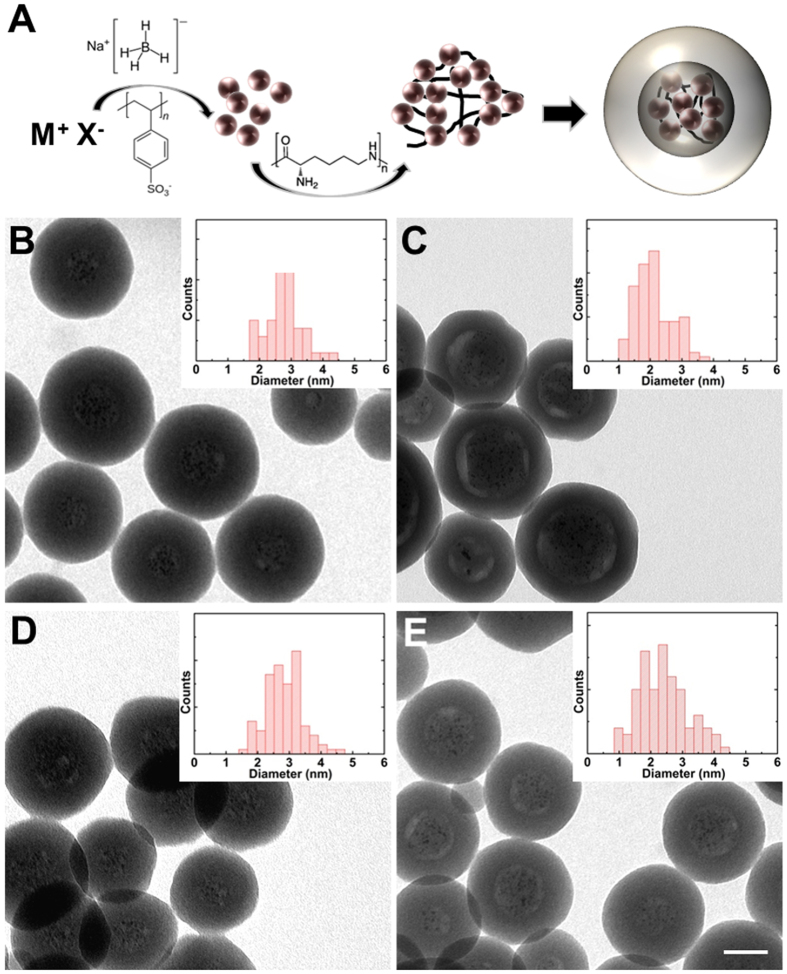
Synthesis of the passion fruit-like nano-architectures (MSi). Scheme of each synthetic step (**A**) and TEM images of AuSi (**B**), PtSi (**C**), AgSi (**D**) and AuPtSi (**E**). Scale bar: 50 nm. The insets show the size distribution histograms of metal NPs in the arrays inside the inner cavity of nano-architectures made on ~100 particles by TEM imaging.

The synthetic steps are schematically reported in Fig. 1A. Metal salts underwent fast reduction by sodium borohydride in the presence of poly(sodium 4-styrene sulfonate) (PSS) resulting in negatively charged metal NPs less than 3 nm in diameter (Supplementary Table S1). NPs were then assembled in spherical arrays by a controlled aggregation achieved by ionic interaction with positive poly(L-lysine) (PL) 15–30 kDa. The arrays were purified by cycles of centrifugation and silica-coated by employing a modified Stöber method, in order to obtain nano-architectures (MSi) of generally 100 nm in diameter and 20 nm of wall thickness, and containing 1–10% w/w of metal (Supplementary Table S1, Table S2, and Fig. S1). TEM images of the nanocapsules are shown in Fig. 1B (AuSi), 1 C (PtSi), 1D (AgSi) and 1E (AuPtSi). The insets report the size dispersion of at least 100 metal NPs collected by TEM (a comprehensive comparison between the size of metal NPs is reported in Fig. S2). Usually, every synthesis results in about 1.5 mg of freeze-dried product and the protocol is scalable to at least 15 mg/synthesis. It is worth to notice that an optimized ratio between NPs, PL and tetraethyl orthosilicate (TEOS, used in the Stöber protocol) was employed for each type of nano-architecture, in order to avoid the formation of large aggregates or empty silica shells. MSi with multiple metals in their central cavity were obtained by mixing the same amounts of colloidal solutions of different metal NPs before aggregation by PL. Standard ammonia-catalysed hydrolysis of TEOS was employed for the synthesis of AuSi, PtSi and AuPtSi nanoparticles. This protocol was not applied to AgSi NPs since silver strongly react with ammonia, leading to the formation of diamminesilver(I) complex ion^23^. Indeed, attempts of silica composition around silver NPs arrays in presence of ammonia resulted in a mix of empty silica shells and big silver NPs outside the shell (Supplementary Fig. S3). AgSi NPs were successfully synthesized in presence of dimethylamine (DMA) as hydrolysis catalyst^23^. It is worth to notice that DMA does not affect the final structure of nano-architectures (Fig. 1D) and thus the reproducibility of the synthetic strategy is preserved. These results confirmed that the success of the protocol is not linked to the kind of metal nanoparticles but only on the simultaneous presence of both PL and PSS in the polymeric arrays. Starting from this evidence, we have also employed our protocol to produce metal-free hollow silica nanospheres (Supplementary Fig. S4) in one-step by performing the silication on polymeric arrays composed by only PL and PSS. Interestingly, moieties such as fluorophores or active agents can be covalently loaded in their cavities by standard coupling reaction on PL before the formation of the nano-architectures^20^,^21^.

The general mechanism of silica shell formation was studied by TEM during the synthetic process. Briefly, we have recorded TEM images every 15 minutes from the addition of the polymeric arrays to the reaction solution (Supplementary Fig. S5). In the general Stöber reaction, the polymerization of orthosilicic acid (resulted from TEOS hydrolysis) occurs when the concentration exceeds the saturation limit in ethanol^24^. The process yields in sequence low- to high-molecular polymers, and, due to condensation, particles of 1–2 nm in size. Then nuclei increase in size following a LaMer growth pattern until their diameter reaches a critical value of 5–7 nm, after which they start to aggregate to form silica nanoparticles^24^. In our protocol the orthosilicic acid is adsorbed in the metal-polymeric arrays before/during its polymerization (15 minutes). Then, silica particles of 1–2 nm formed in solution, aggregate on the external surface of the arrays (30 minutes) and the remaining orthosilicic acid continues to form a complete shell until its saturation limit is reached or the reaction interrupted (usually 3 h). It is interesting to notice that: i) the concentration of gold nanoparticles appears to decrease during the capsules formation and ii) the silica shell of the complete nano-architectures does not show gold nanoparticles inclusion. These findings suggest that a part of gold nanoparticles is ejected during the silica shell densification. As expected, a minimum TEOS quantity threshold must be supplied in order to complete the reaction and produce hollow structures (Supplementary Fig. S4).

**Figure 2:**
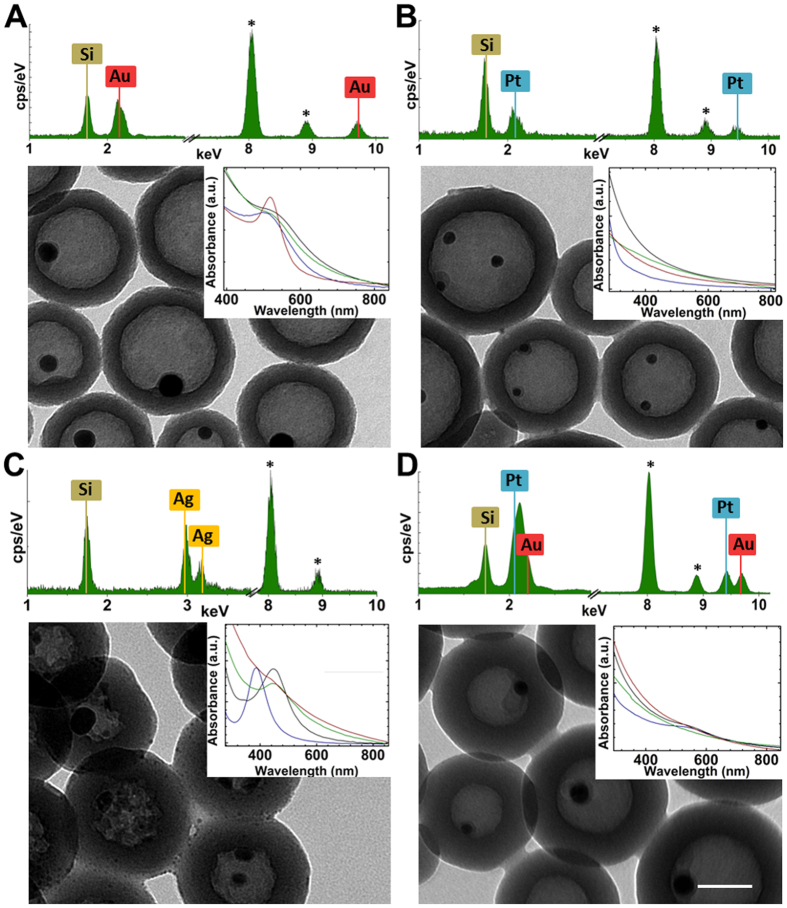
Calcinated MSi nanostructures. TEM images of calcinated AuSi (**A**), PtSi (**B**), AgSi (**C**) and AuPtSi (**D**). Scale bar 50 nm. The insets show the absorbance spectra of metal NPs (blue), metal NPs arrays (black), MSi (green) and calcinated MSi (red), respectively. EDX spectra are reported on top of TEM images for each sample. Copper peaks from TEM grid are marked with a black star.

Calcination of MSi produces yolk-shell nanostructures in which naked metal NPs are confined inside the silica shells (Fig. 2). During the process the polymers are burned, and ultrasmall metal NPs are melted and re-condensate in naked nanospheres with diameters ranging from 10–30 nm, depending on the MSi (Supplementary Table S1 and Fig. S2). Nano-structured metals have a lower melting point with respect to bulks, thus it is not surprising their sintering during the calcination process^25^. The removal of the organic compounds was also confirmed by multipoint Energy Dispersive X-ray (EDX) analyses (Supplementary Fig. S6), which show that the peak of sulphur from PSS disappears from the calcinated nanostructures. Gases are able to cross silica walls even if they are dense and over 20 nm^26^, thus we suggest that, during the calcination, sulphur turns into SOx gases and escapes from nano-architectures. It is expected that also carbon and nitrogen from both PSS and PL experience the same occurrence, but their EDX peaks in MSi are hidden by the carbon of the TEM grid. EDX analyses (Supplementary Fig. S6) from scanned areas far from the metal core of the nano-architectures also confirm that no migration of metals on their internal/external surfaces occurred. This is also confirmed by the correlation between the density of NPs before the calcination and the size of the calcinated metal nanostructures inside nano-architectures (Supplementary Fig. S4).

Calcination of AuSi at 600 °C resulted in silica shells with a single metal core in their central cavity (TEM image, Fig. 2A), while calcinated PtSi at the same temperature are mainly composed by multinucleated cores of usually two nanoparticles (TEM image, Fig. 2B). This can be ascribed to the higher resistance of platinum NPs over sintering and coalescence^27^. Consistently, calcinations of AuSi at 350 °C produced multinucleated nano-architectures with cores of less than 10 nm in diameter (Supplementary Fig. S7). Thus, we speculate that calcinations at temperatures higher than 600 °C would likely bring to single cored nanostructures also for PtSi. Sintering of AgSi at a temperature of 600 °C could lead to direct solid-to-gas transition of the metal. Indeed, it was demonstrated that 3 nm silver NPs undergo sublimation at 600–700 °C^28^. Thus, in order to avoid silver sublimation, the calcination process of AgSi was carried out at a lower temperature (350 °C) in a sand bath on a hotplate, yielding mostly single-cored nanostructures (Fig. 2C). Interestingly, AuPtSi are mainly mononucleated (Fig. 2D) if calcinated at 600 °C and EDX point analysis centred on the metal nuclei showed that each metal core is a gold/platinum mixture (Supplementary Fig. S8) with a probable core/shell structure^29^.

The optical behaviours of the nano-architectures were investigated by absorbance measurements and are reported in the insets of Fig. 2. The discussion on the spectral features of AuSi is reported elsewhere^20^. The spectra of spherical platinum nanoparticles usually show a strong extinction in the UV region, which red-shifts up to the visible region only when the diameter of the NPs exceeds 80 nm^30^. Indeed, the extinction spectra of 3 nm Pt seeds, NPs arrays, PtSi and calcinated PtSi, (Fig. 2B, inset) do not show any sharp peak in the visible region but only a broad tail. The spectra of Ag NPs in the various configuration (Fig. 2C, inset) is consistent with what reported in our previous work^20^ for AuSi: the plasmon band of 2.8 ± 0.6 nm silver NPs (blue) is localized at 380 nm and it is subjected to a remarkable red-shift up to 440 nm upon aggregation into spherical arrays by PL (black). This behaviour is related to the close proximity, in the arrays, of Ag NPs to each other^31^, thus they are optically coupled and behave like a single bigger NP. As expected, the spectral shift for Ag NPs is larger than the one reported for gold ones^32^. The absorbance maximum wavelength slightly varies when the silica shell is composed, and the spectrum presents an increased Rayleigh scattering background. When nano-architectures were calcinated, the plasmon bands remained stable, underlying that arrays of strictly packed ultrasmall nanoparticles effectively mimic the optical behaviour of single bigger NPs with the same final volume. The absorbance spectra from colloidal solution of mixed Au and Pt ultrasmall NPs (Fig. 2D, inset) show the typical plasmon band of gold at around 520 nm (blue) which remains substantially unaltered after aggregation by PL (black). Interestingly, the plasmon band of gold completely disappears after the formation of the silica shell (green) and also upon calcination (red). This can be related to both: i) the decrease of the contribute on the spectra of the plasmon peak with respect to the Rayleigh scattering, and ii) to a thermally dependent phase segregation, in which the two metals are apart in a core (Au)/shell (Pt) structure^33^,^34^.

Yolk-shell nano-architectures have several advantages over conventional catalysts, such as increased activity, recyclability and improved stability^22^,^35^. On the other hand, the active part of the catalyst is confined inside a silica shell. Thus, it could be not well accessible from the reactive, resulting in a decrease of the overall catalytic activity in some reactions^36^. However, it is worth to notice that by tuning the pores of the shell it is possible to partially overcome the activity loss by maintaining the advantage of avoiding the aggregation of nanostructures during the reaction. In our nano-architectures, the aggregation shielding is jointly combined with a completely naked surface of the metal nanoparticles, produced by a simple, versatile and inexpensive protocol.

The permeability of MSi and calcinated MSi was investigated on AuSi by their treating with potassium cyanide (KCN) solutions. KCN is able to penetrate silica shells and convert gold nanoparticles to a water-soluble aurocyanide coordination complex, [Au(CN)_2_]^−^, without affecting the structure of the nano-architectures (Supplementary Fig. S9)^37^. We observed that water-solution of AuSi lose their typical extinction band after the KCN treatment, confirming the mesoporous nature of the silica shell. On the other hand, gold nanoparticles inside calcinated MSi remain intact, suggesting a reduction of the silica shell permeability after the calcination, despite the processing temperatures are well below the glass transition temperature of silica^38^.

**Figure 3:**
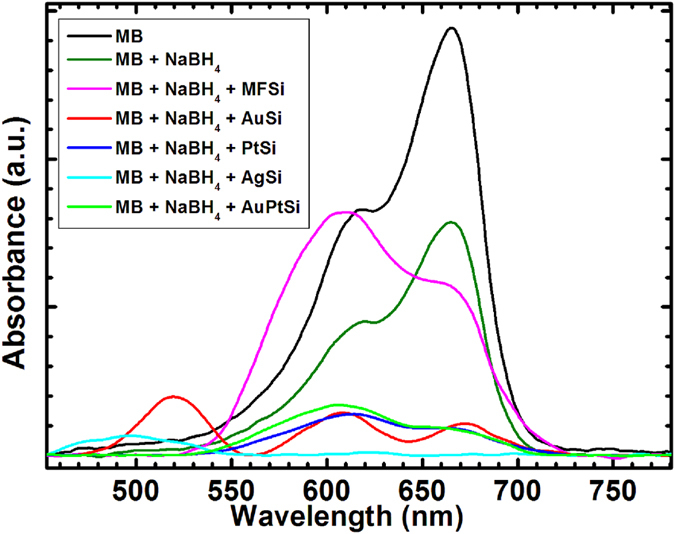
UV/Vis absorbance spectra of Methylene Blue (MB) (black) and 15 minutes after the addition of sodium borohydride (olive) in presence of MFSi (magenta), calcinated AuSi (red), PtSi (blue), AgSi (cyan) and AuPtSi (green). Background subtraction was applied to all spectra.

As proof of concept, the potential of our nano-architectures as effective catalysts was evaluated in the reduction of the cationic dye methylene blue (MB) by sodium borohydride at ambient conditions^39^,^40^. The catalytic activity of calcinated MSi was monitored by recording the absorbance spectra of MB in aqueous solution for 15 minutes after the addition of sodium borohydride (Fig. 3). As expected, the reduction of MB results in a decrease of its absorbance band at 664 nm^39^,^40^. However, the decrease is far more pronounced when calcinated MSi are added to the mix, indicating a net acceleration in the rate of reduction of the dye (Fig. 3). In agreement with other reports, the better performance for this type of reaction is recorded for the silver-based calcinated nano-architectures^41^. It is interesting to notice the pronounced inversion between the band at 664 nm and the shoulder at 612 nm in presence of MFSi (Fig. 3), that can be ascribed to a dimerization of MB on the silica surface^42^.

Remarkably, no significant catalytic effect is observed in presence of MFSi (Fig. 3, magenta) confirming that the catalytic activity is related by the metal cores of calcinated nano-architectures.

## Conclusion

In summary, we described a highly reproducible and robust protocol to synthesize biodegradable nano-architectures with sundry noble metals of interest. The synthetic route yields nanocapsules which have the promising to be effective theranostics devices which are biodegraded in less than 48 h in potentially kidney-clearable constituents. These modular nanosystems provide a high level of versatility thanks to the possibility to conjugate the polymers inside the cavity and the outer surface with drugs, dyes, targeting agents and complexing molecules by standard protocols. Upon calcination, they have the potential to be effective catalytic systems due to the bare surface of metal cores stabilized by a thin silica shell. Moreover, these nanosystems could be useful in composition of photonic crystals with peculiar optical features. Notably, this strategy was extended to metal-free hollow silica nanospheres built in a single-step synthesis over templates that do not need removal, and which can be covalently conjugated to drug and dye moieties.

## Methods

### Au

#### Synthesis of AuNPs

Au NPs with a diameter of approximately 3 nm were prepared according to the following procedure. To 20 mL of milliQ water were added 10 μL of poly(sodium 4-styrene sulfonate) 70 kDa (30% aqueous solution) and 200 μL of HAuCl_4_ aqueous solution 25 mM. During vigorously stirring, 200 μL of NaBH_4_ (4 mg/mL in milliQ water) were added quickly, and the mixture was stirred vigorously for other 2 minutes. After the addition of NaBH4, the solution underwent some color changes until becoming brilliant orange. Before its use the solution was generally aged for at least 30 minutes and employed without further purification.

#### Synthesis of AuNPs arrays

To 20 mL of AuNPs solution were added 200 μL of poly(L-lysine) hydrobromide 15–30 kDa milliQ solution (20 mg/mL) and the mixture was allowed to stir for 30 minutes at room temperature. The as synthesized AuNPs aggregates were collected by centrifugation (13400 rpm for 3 minutes), suspended in 2 mL of milliQ water and sonicated for maximum 4 minutes.

#### Synthesis of AuSi

In a 100 mL round bottomed flask were added 70 mL of absolute ethanol followed by 2.4 mL of ammonium hydroxide solution (30% in water), and 40 μL of tetraethyl orthosilicate (TEOS, 98%). The solution was allowed to stir for 20 minutes at RT. 2 mL of the AuNPs arrays previously prepared were added to the reaction flask and the solution was allowed to stir for further 3 h at RT. The as-synthesized AuSi were collected by 30 minutes centrifugation at 4000 rpm, washed twice with ethanol to remove unreacted precursors and suspended in 1 mL of ethanol. A short spin centrifugation was employed in order to separate the structure over 150 nm from the supernatant, which was recovered as a pink- iridescent solution. The solution was centrifuged at 13400 rpm for 5 minutes, suspended in 500 μL milliQ water, sonicated for 5 minutes and freeze-dried overnight. Usually, about 1.5 mg of a brilliant pink powder is obtained, which remains stable for at least 1 year if stored sealed in the dark at 10 °C.

#### Synthesis of calcinated AuSi

The pink powder of AuSi was calcinated in a standard furnace following the sequence: 200 °C for 2 h, 400 °C for 1 h and 600 °C for 2 h. The resulting powder undergoes a colour change turning into violet depending on the formation of a single gold nanostructure of 20–30 nm inside the hollow silica shell.

### Pt

#### Synthesis of PtNPs

Pt NPs with a diameter of approximately 3 nm were prepared according to the following procedure. To 20 mL of milliQ water were added 20 μL of poly(sodium 4-styrene sulfonate) 70 kDa (30% aqueous solution) and 300 μL of H_2_PtCl_6_ aqueous solution 25 mM. During vigorous stirring, 200 μL of NaBH4 (4 mg/mL in milliQ water) were added quickly, and the mixture was stirred vigorously for other 2 minutes. After the addition of NaBH4, the solution underwent some colour changes until becoming brownish yellow. Before its use the solution was generally aged for at least 30 minutes and employed without further purification.

#### Synthesis of PtNPs arrays

To 20 mL of PtNPs solution were added 200 μL of poly(L-lysine) hydrobromide 15–30 kDa milliQ solution (20 mg/mL) and the mixture was allowed to stir for 30 minutes at room temperature. The as synthesized PtNPs aggregates were collected by centrifugation (13400 rpm for 3 minutes), suspended in 2 mL of milliQ water and sonicated for maximum 4 minutes.

#### Synthesis of PtSi

In a 100 mL round bottomed flask were added 70 mL of absolute ethanol followed by 2.4 mL of ammonium hydroxide solution (30% in water), and 40 μL of tetraethyl orthosilicate (TEOS, 98%). The solution was allowed to stir for 20 minutes at RT. 2 mL of the PtNPs arrays previously prepared were added to the reaction flask and the solution was allowed to stir for further 3 h at RT. The as-synthesized PtSi were collected by 30 minutes centrifugation at 4000 rpm, washed twice with ethanol to remove unreacted precursors and suspended in 1 mL of ethanol. A short spin centrifugation was employed in order to separate the structure over 150 nm from the supernatant, which was recovered as a dark grey solution. The solution was centrifuged at 13400 rpm for 5 minutes, suspended in 500 μL milliQ water, sonicated for 5 minutes and freeze-dried overnight. Usually, about 1.5 mg of a dark grey powder is obtained, which remains stable for at least 1 year if stored sealed in the dark at 10 °C.

#### Synthesis of calcinated PtSi

The dark grey powder of PtSi was calcinated in a standard furnace following the sequence: 200 °C for 2 h, 400 °C for 1 h and 600 °C for 2 h. The resulting powder undergoes a colour change turning into light gray depending on the formation of a multiple platinum nanostructures of 10–20 nm inside the hollow silica shell.

### Bimetallic AuPt

#### Synthesis of AuPtNPs arrays

10 mL of AuNPs and 10 mL of PtNPs were mixed and 200 μL of poly(L-lysine) hydrobromide 15–30 kDa milliQ solution (20 mg/mL) were added to the mixture, which was allowed to stir for 30 minutes at room temperature. The as synthesized AuPtNPs aggregates were collected by centrifugation (13400 rpm for 3 minutes), suspended in 2 mL of milliQ water and sonicated for maximum 4 minutes.

#### Synthesis of AuPtSi

In a 100 mL round bottomed flask were added 70 mL of absolute ethanol followed by 2.4 mL of ammonium hydroxide solution (30% in water), and 40 μL of tetraethyl orthosilicate (TEOS, 98%). The solution was allowed to stir for 20 minutes at RT. 2 mL of the AuPtNPs arrays previously prepared were added to the reaction flask and the solution was allowed to stir for further 3 h at RT. The as-synthesized AuPtSi were collected by 30 minutes centrifugation at 4000 rpm, washed twice with ethanol to remove unreacted precursors and suspended in 1 mL of ethanol. A short spin centrifugation was employed in order to separate the structure over 150 nm from the supernatant, which was recovered as a plum solution. The solution was centrifuged at 13400 rpm for 5 minutes, suspended in 500 μL milliQ water, sonicated for 5 minutes and freeze-dried overnight. Usually, about 1.5 mg of a plum powder is obtained, which remains stable for at least 1 year if stored sealed in the dark at 10 °C.

#### Synthesis of calcinated AuPtSi

The plum powder of AuPtSi was calcinated in a standard furnace following the sequence: 200 °C for 2 h, 400 °C for 1 h and 600 °C for 2 h. The resulting powder undergoes a color change turning into light gray depending on the formation of a single AuPt alloy nanostructure of 10–20 nm inside the hollow silica shell.

### Ag

#### Synthesis of AgNPs

AgNPs with a diameter of approximately 3 nm were prepared according to the following procedure. To 20 mL of 4 °C milliQ water were added 10 μL of poly(sodium 4-styrene sulfonate) 70 kDa (30% aqueous solution) and 200 μL of AgNO_3_ aqueous solution 25 mM. During vigorous stirring, 200 μL of NaBH_4_ (8 mg/mL milliQ water) were added quickly, and the mixture was stirred vigorously for other 2 minutes and employed immediately for aggregation by poly(L-lysine).

#### Synthesis of AgNPs arrays

To 20 mL of AgNPs were added 200 μL of poly(L-lysine) hydrobromide 15–30 kDa milliQ solution (20 mg/mL). The solution turned to a dark yellow colour and the was allowed to stir for 30 minutes at room temperature. The as synthesized AgNPs aggregates were collected by centrifugation (13400 rpm for 3 minutes), suspended in 2 mL of milliQ water and sonicated for maximum 4 minutes.

#### Synthesis of AgSi

In a 100 mL round bottomed flask were added 70 mL of absolute ethanol and 2.4 mL of milliQ water, followed by the addition of 2 mL of the AgNPs arrays previously prepared and 40 μL of tetraethyl orthosilicate (TEOS, 98%). After 5 min, 200 μL of dimethylamine solution (30% in ethanol) were added and the solution was allowed to stir for 3 h at RT. The as-synthesized AgSi were collected by 30 minutes centrifugation at 4000 rpm, washed twice with ethanol to remove unreacted precursors and suspended in 1 mL of ethanol. A short spin centrifugation was employed in order to separate the structure over 150 nm from the supernatant, which was recovered as a dark yellow solution. The solution was centrifuged at 13400 rpm for 5 minutes, suspended in 500 μL milliQ water, sonicated for 5 minutes and freeze-dried overnight. Usually, about 1.5 mg of a dark yellow powder is obtained, which remains stable for at least 1 year if stored sealed in the dark at 10 °C.

#### Synthesis of calcinated AgSi

The dark yellow powder of AgSi was calcinated at 350 °C for 2 h in a sand bath on a hotplate. The resulting powder undergoes a colour change turning into light yellow depending on the formation of a single Ag nanostructure of 10–20 nm inside the hollow silica shell.

### Synthesis of Metal-free nanostructures (MFSi)

To 20 mL milliQ were added 10 μL of poly(sodium 4-styrene sulfonate) 70 kDa (30% aqueous solution) and 200 μL of poly(L-lysine) hydrobromide 15–30 kDa milliQ solution (20 mg/ml) during ultrasonication. After 1 minute the mixture was left under stirring for 15 minutes at RT and then centrifuged (13400 rpm for 3 minutes). The white precipitate was recovered with 2 mL milliQ water and added to a 100 mL round bottomed flask containing a mixture of 70 mL of absolute ethanol, 2.4 mL of ammonium hydroxide (30% in water) and 40 μL of tetraethyl orthosilicate (TEOS, 98%). The mixture was allowed to stir for 3 h at RT. The as-synthesized MFSi were collected by 30 minutes centrifugation at 4000 rpm, washed twice with ethanol to remove unreacted precursors and suspended in 1 mL of ethanol. A short spin centrifugation was employed in order to separate the structure over 150 nm from the supernatant, which was recovered as a milky white solution. The solution was centrifuged at 13400 rpm for 5 minutes, suspended in 500 μL milliQ water, sonicated for 5 minutes and freeze-dried overnight. Usually, about 1.5 mg of a white powder is obtained, which remains stable for at least 1 year if stored sealed in the dark at 10 °C.

### UV/Vis spectrophotometry

Extinction spectra were collected by means of a double beam spectrophotometer Jasco V-550 UV/VIS equipped with quartz cuvettes of 1.5 mm path length and normalized to the maximum absorbance. MilliQ water was employed as solvent.

### Electron microscopy

TEM observations of nanoparticles: measurements were carried out on a ZEISS Libra 120 TEM operating at an accelerating voltage of 120 kV, equipped with an in-column omega filter. The colloidal solutions were deposited on 300-mesh carbon-coated copper grids and air dried overnight before being imaged.

### EDX Point analysis

Energy dispersive X-ray spectroscopy (EDX) analysis were performed on the same microscope, working in scanning mode (STEM), thanks to a Bruker XFlash^®^ 6 T | 60 SDD detector. A beam spot size of 5 nm was used on the smallest NPs. On the biggest one a spot up to 20 nm could be used to have a better signal.

### ICP-MS Analysis

Nanoparticles were dissolved in 1 mL aqua regia (prepared with ICP-MS grade HCl and HNO_3_) and digested under microwave irradiation (200 °C/15 minutes) in Teflon-lined vessels. The resulting solution was diluted to 10 mL with ICP-MS grade water, and content of Ag, Pt and Au was determined by ICP-MS analysis against a standard calibration curve.

### Gold dissolution

200 μg of freeze-dried sample were dispersed in 1 mL milliQ water and 50 μL of potassium cyanide water solution (10 mg/mL) were added. After 1 h stirring (25 °C, 750 rpm) the sample was precipitated by centrifugation (13400 rpm, 3 min) and redispersed in 1 mL milliQ water. The resulting dispersion was employed for TEM imaging and UV/Vis spectroscopy.

### Methylene Blue reduction

100 μg of freeze dried calcinated MSi or MFSi were added to a solution of Methylene Blue Hydrate (250 μl, 5 μg/mL), followed by the addition of sodium borohydride (25 μL, 0.5 mg/mL). The samples were maintained under stirring at RT and the absorbance spectra recorded every 2 minutes for a maximum of 15 minutes.

## Additional Information

**How to cite this article**: Cassano, D. *et al*. Passion fruit-like nano-architectures: a general synthesis route. *Sci. Rep.* **7**, 43795; doi: 10.1038/srep43795 (2017).

**Publisher's note:** Springer Nature remains neutral with regard to jurisdictional claims in published maps and institutional affiliations.

## Supplementary Material

Supplementary Information
