# Supplementary material for: Passion fruit-like nano-architectures: a general synthesis route
Source: Sci Rep. 2017 Mar 3;7:43795. doi: 10.1038/srep43795 (PMC5335552; doi:10.1038/srep43795)
Supplement: Supplementary Information [file srep43795-s1.pdf]

Supplementary Information

Passion fruit-like nano-architectures: a general synthesis route

Domenico Cassano,[a,b] Jérémy David,[a] Stefano Luin,[b,c] and Valerio Voliani*[a]

[a] Mr. D. Cassano, Dr. J. David, Dr. V. Voliani*

Center for Nanotechnology Innovation @NEST, Istituto Italiano di Tecnologia

Piazza San Silvestro, 12 Pisa (IT) 56127

E-mail: valerio.voliani@iit.it

[b] Mr. D. Cassano, Dr. S. Luin

NEST-Scuola Normale Superiore

Piazza San Silvestro, 12 Pisa (IT) 56127

[c] Dr. S. Luin

NEST, Istituto Nanoscienze - CNR

Piazza San Silvestro, 12 Pisa (IT) 56127

Table S1

MSi	***Size of metal nanoparticles before calcination (nm)***	***Size of metal nanoparticles after calcination (nm)***	***Size of passion fruit–like nano-architectures (nm)***	***Silica shell thickness (nm)***
AuSi	2.78 ± 0.52	23.31 ± 6.33	97,61 ± 8,82	20,24 ± 1,60
PtSi	2.14 ± 0.58	17.26 ± 6.03	106.53 ± 15.11	22.21 ± 1.72
AgSi	2.82 ± 0.56	14.47 ± 2.91	104.97 ± 16.75	19,97 ± 1.95
AuPtSi	2.78 ± 0.52 ; 2.14 ± 0.58	20.51 ± 5.73	96,46 ± 9,51	26.30 ± 2.68

Table S1. Diameters of metal NPs inside the nanocapsules before and after the calcination, diameters of passion fruit-like nano-architectures and average thickness of silica shells. Analyses performed on at least 100 NPs collected by TEM.

Table S2

MSi	***Metal weight percentage***
AuSi	4.6 ± 0.2%
PtSi	1.63 ± 0.4%
AgSi	9.1 ± 0.3%
AuPtSi	3.2 ± 0.2% / 1.4 ± 0.2%

Table S2. ICP-MS analyses of passion fruit-like nano-architectures. The metal weight percentage is referred to the ratio between the weight of metal contained in the nano-architectures and the total weight of the freeze-dried samples. In AuPtSi line are reported the percentage for both, respectively, gold and platinum.

Figure S1

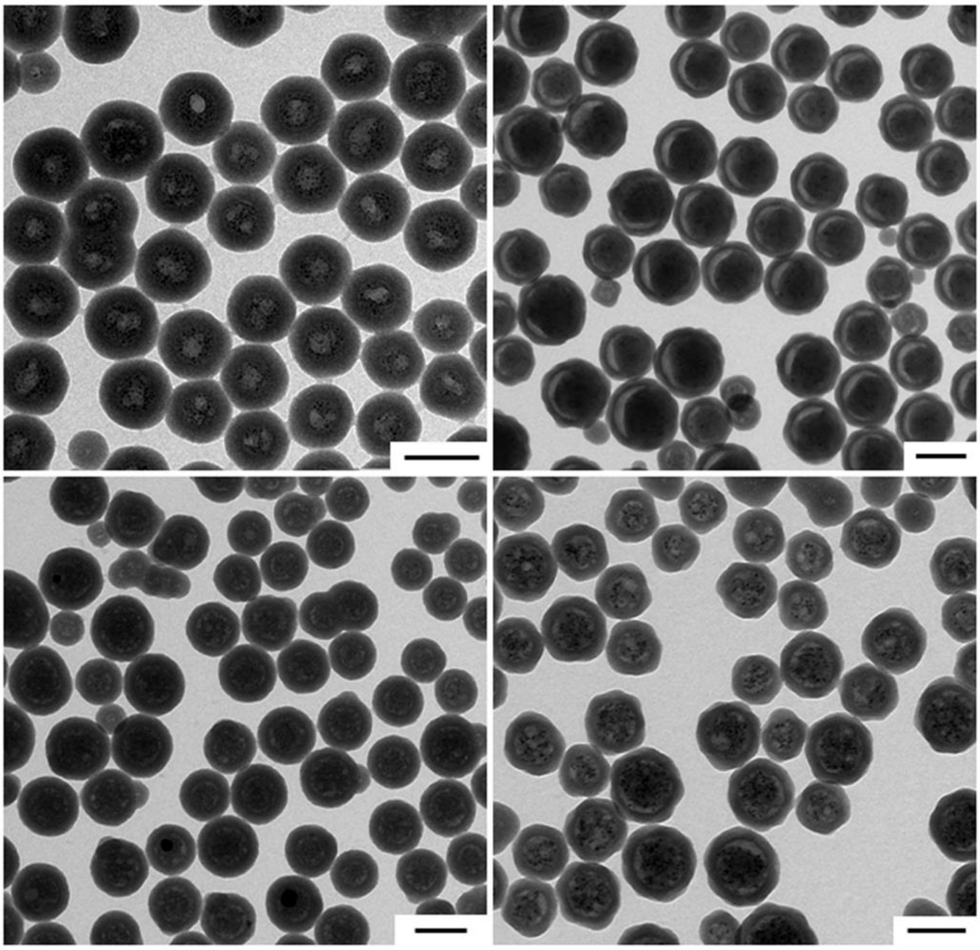

Figure S1. Typical wide area TEM images of (clockwise) AuSi, PtSi, AgSi, AuPtSi. Scalebars: 100 nm.

Figure S2

A

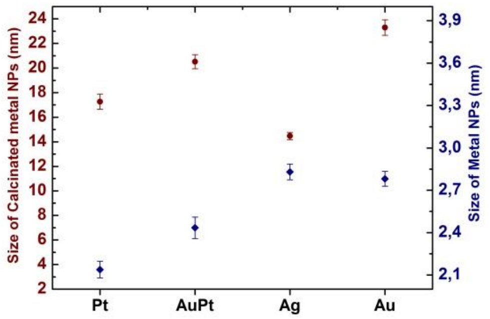

B

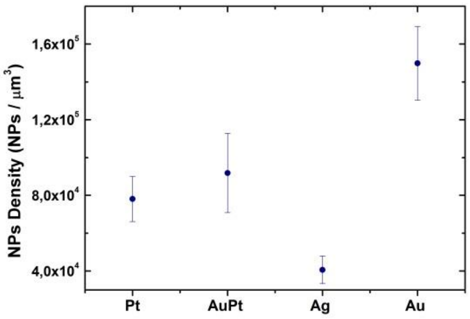

Figure S2. A) Size of metal nanoparticles inside the nano-architectures before (blue) and after (red) calcination. The count is based on at least 50 nano-architectures. B) Density of ultrasmall metal nanoparticles in the nano-architectures. The count is based on minimum 50 nano-architectures, the considered volume is the entire volume of the nano-architecture.

Figure S3

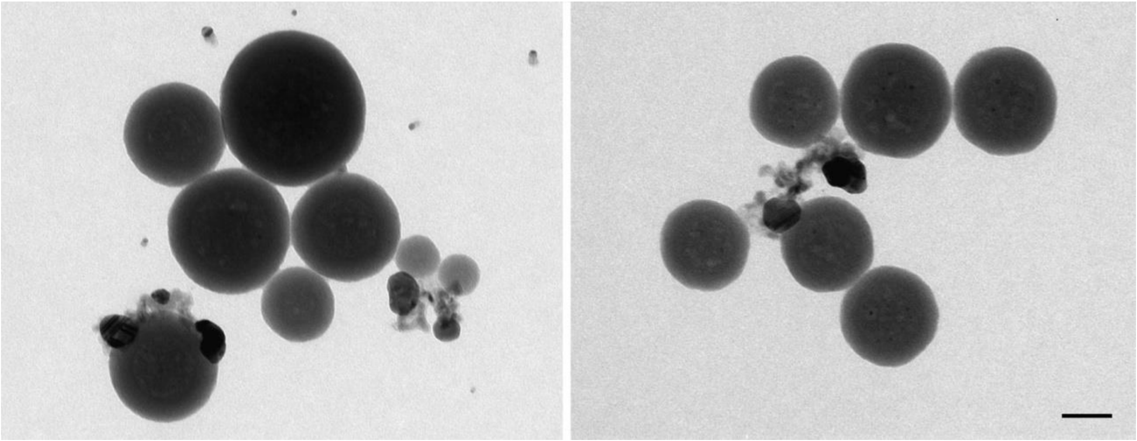

Figure S3. TEM images of AgSi synthesized in the presence of ammonia. The synthesis yields nearly hollow silica nanocapsules with silver nanoparticles of about 30 nm outside the shells. Scale bar 50 nm.

Figure S4

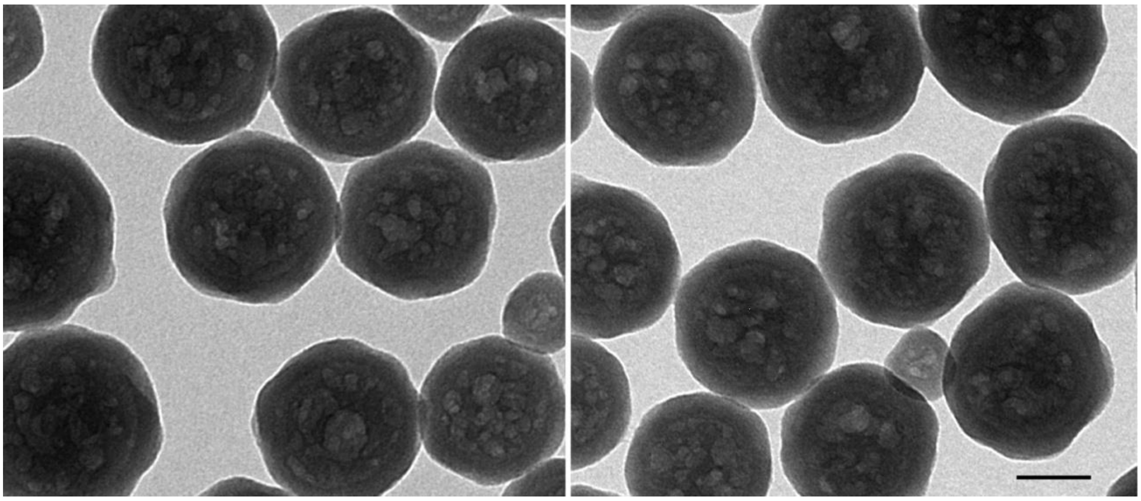

Figure S4. TEM images of metal-free nano-architectures (MFSi). Scale bar 50 nm.

Figure S5

A

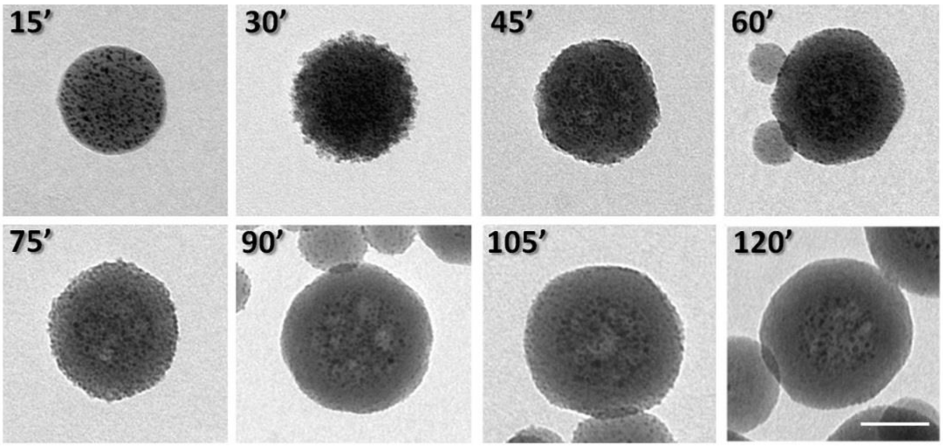

B

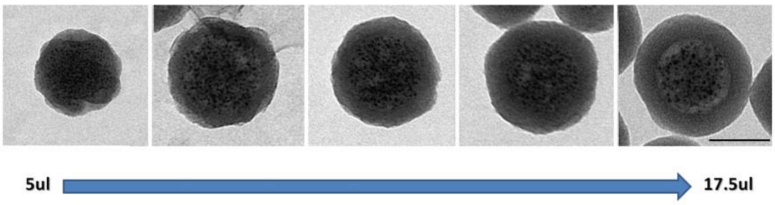

Figure S5. A) Typical TEM images taken every 15’ during the AuSi formation. Scalebar 60 nm. B) TEM images of AuSi nanoparticles after 2h reaction by varying the concentration of TEOS. Scalebar 60 nm.

Figure S6

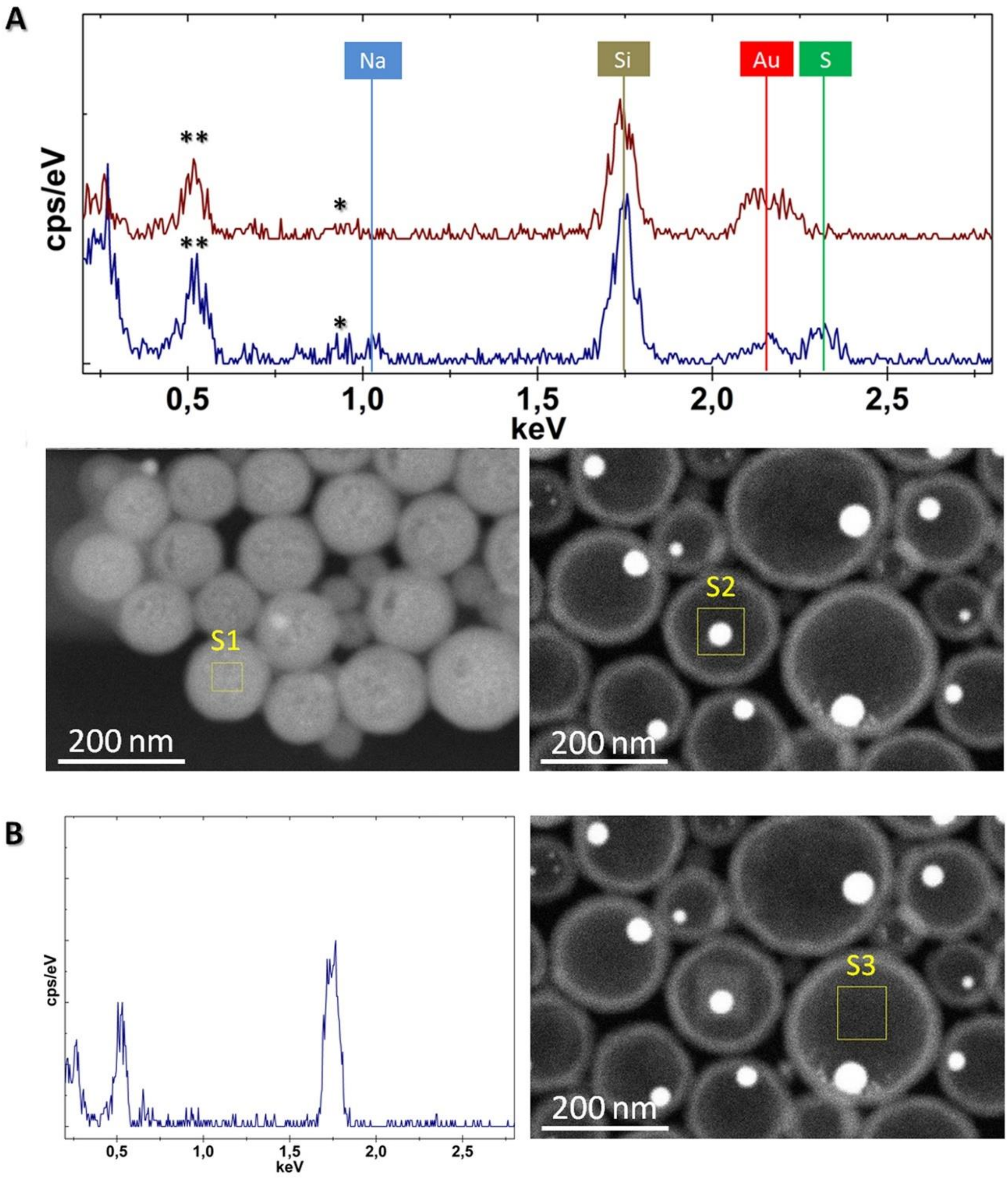

Figure S6. A) Upper panel: EDX multipoint spectra of AuSi (blue) and calcinated AuSi (red). Copper from TEM grids and oxygen sample holder are marked with single and double star, respectively. Bottom: STEM images of AuSi (left) and calcinated AuSi (right) with EDX scanned area marked with yellow squares. B) EDX spectrum of calcinated AuSi (left) from a scanned area far from the metal core (STEM image, right) confirms that the peak of sulphur is absent and not overwhelmed by the peak of gold.

Figure S7

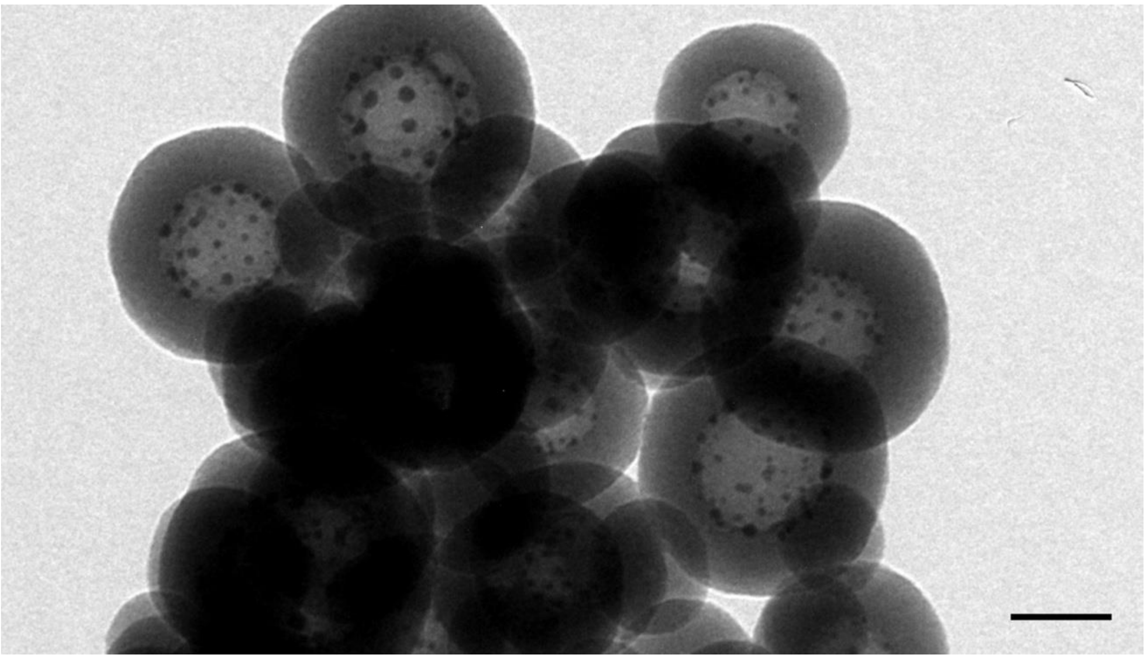

Figure S7. TEM image of AuSi after calcination at 350 °C showing multinucleated nanostructures. Scalebar 50nm.

Figure S8

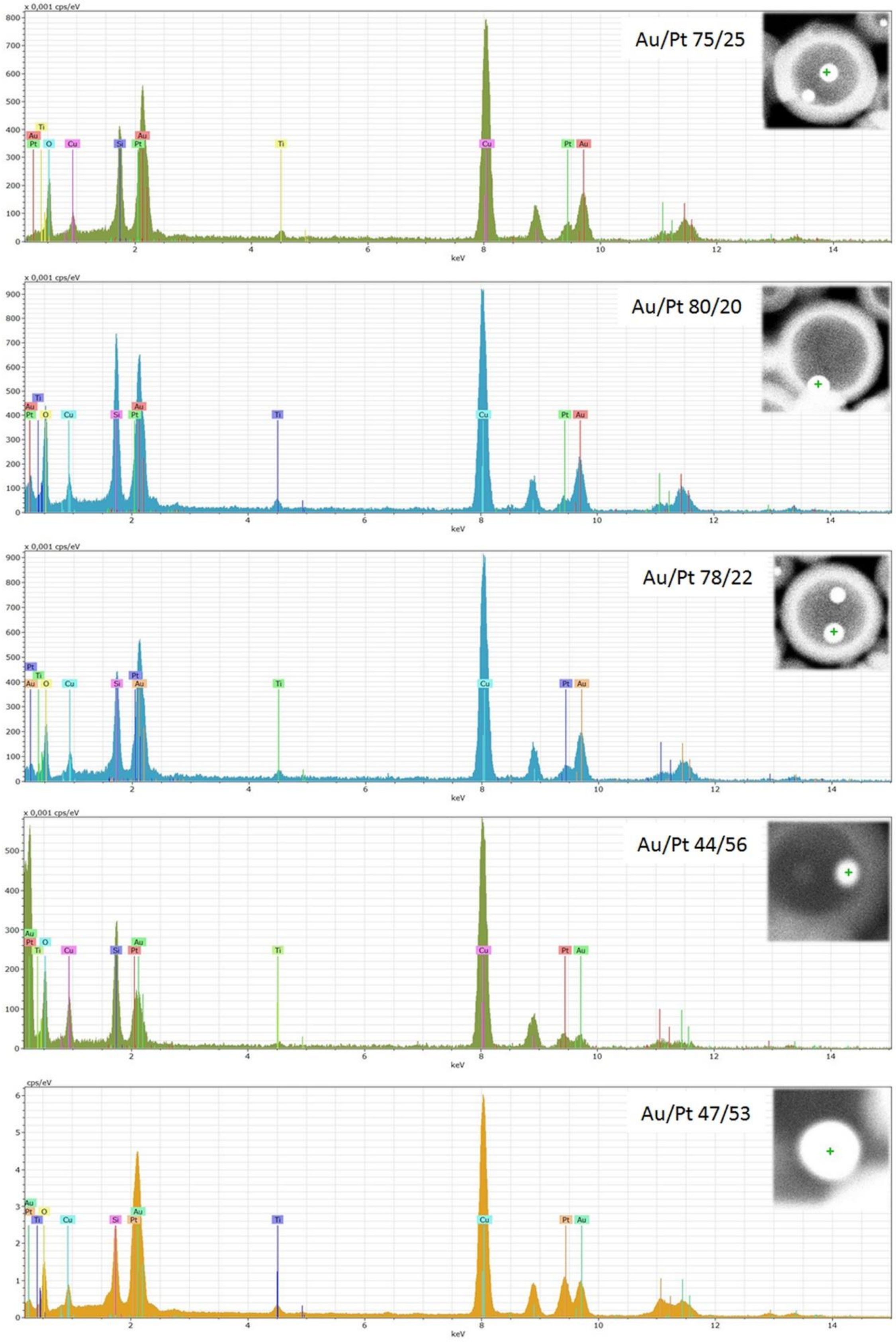

Figure S8. EDX quantitative analyses of AuPtSi calcinated samples. Inset show STEM images of metal cores on which EDX point analyses were performed and the percentages of Au/Pt contents.

Figure S9

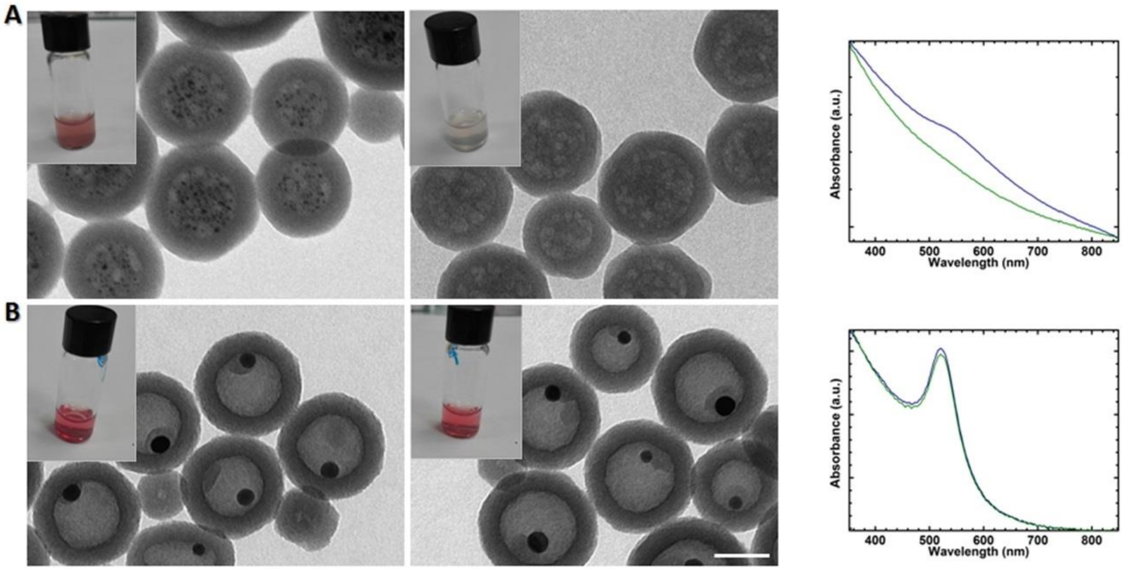

Figure S9. (A) TEM images of AuSi before (left) and after (center) KCN treatment, and the relative absorbance spectra (right, blue and green lines, respectively). (B) TEM images of calcinated AuSi before (left) and after (center) KCN treatment, and the relative absorbance spectra (right, blue and green lines, respectively). Insets show colloidal solutions in milliQ water. Scale bar 50nm.
